# Rasch scaling procedures for informing development of a valid Fetal Surveillance Education Program multiple-choice assessment

**DOI:** 10.1186/1472-6920-9-20

**Published:** 2009-04-29

**Authors:** Nathan Zoanetti, Patrick Griffin, Mark Beaves, Euan M Wallace

**Affiliations:** 1 Assessment Research Centre, Melbourne Graduate School of Education, University of Melbourne, Australia; 2 Royal Australian and New Zealand College of Obstetricians and Gynaecologists, Australia; 3 Department of Obstetrics and Gynaecology, Monash Institute of Medical Research, Monash University, Australia

## Abstract

**Background:**

It is widely recognised that deficiencies in fetal surveillance practice continue to contribute significantly to the burden of adverse outcomes. This has prompted the development of evidence-based clinical practice guidelines by the Royal Australian and New Zealand College of Obstetricians and Gynaecologists and an associated Fetal Surveillance Education Program to deliver the associated learning. This article describes initial steps in the validation of a corresponding multiple-choice assessment of the relevant educational outcomes through a combination of item response modelling and expert judgement.

**Methods:**

The Rasch item response model was employed for item and test analysis and to empirically derive the substantive interpretation of the assessment variable. This interpretation was then compared to the hierarchy of competencies specified a priori by a team of eight subject-matter experts. Classical Test Theory analyses were also conducted.

**Results:**

A high level of agreement between the hypothesised and derived variable provided evidence of construct validity. Item and test indices from Rasch analysis and Classical Test Theory analysis suggested that the current test form was of moderate quality. However, the analyses made clear the required steps for establishing a valid assessment of sufficient psychometric quality. These steps included: increasing the number of items from 40 to 50 in the first instance, reviewing ineffective items, targeting new items to specific content and difficulty gaps, and formalising the assessment blueprint in light of empirical information relating item structure to item difficulty.

**Conclusion:**

The application of the Rasch model for criterion-referenced assessment validation with an expert stakeholder group is herein described. Recommendations for subsequent item and test construction are also outlined in this article.

## Background

It is widely recognised that a significant number of adverse obstetric outcomes continue to arise from inappropriate use or interpretation of intrapartum fetal surveillance [1-4]. In both the United Kingdom and Australia, national reports on perinatal morbidity and mortality have highlighted that deficiencies in fetal surveillance practice continue to contribute significantly to the burden of adverse outcomes [2,4]. This has led to a call for regular training in fetal surveillance for all staff involved in the care of women in labour [1].

In light of these national reports and the UK recommendations, it is perhaps surprising that options for formal education and credentialing in fetal surveillance remain limited. A recent survey of education and credentialing practices at public maternity hospitals in Victoria reported that only 33% had an existing intrapartum fetal surveillance education program in place and only 10% had some form of credentialing [5].

As a first step to improving quality of care in this setting, the Royal Australian and New Zealand College of Obstetricians and Gynaecologists (RANZCOG) developed, with funding from the public hospital insurer – Victorian Managed Insurance Authority (VMIA), evidence-based clinical practice guidelines on intrapartum fetal surveillance [6]. It was hoped that these guidelines would become a valued resource for maternity care providers and that they might facilitate both standardisation of existing local education programs and the development of new resources. However, it was also recognised that the development of guidelines alone, unsupported by education, would be unlikely to have significant impact on clinical practice. Accordingly, in 2004 the RANZCOG developed an education and credentialing program based upon the Clinical Practice Guidelines. It was anticipated that the proposed program would decrease perinatal morbidity and mortality attributable to intrapartum fetal asphyxia by:

1. improving the knowledge of the underlying pathophysiology of all health professionals undertaking fetal surveillance

2. improving the skills of clinicians (midwives and doctors) providing maternity care

3. improving the appropriate use and interpretation of intrapartum fetal surveillance, including intermittent auscultation and continuous electronic fetal monitoring

4. developing a credentialing program in fetal surveillance based on a core set of competencies

This education program was introduced in 2005 and has so far had in excess of 9000 participants. The evaluation of the program by participants has been extremely positive to date [7].

### Assessment of core competencies in fetal surveillance

While the education program has been well-received by clinicians, one of the primary aims was also to develop an associated credentialing program. As yet, there is no formally validated approach to assessing practitioner knowledge in fetal surveillance in the Australia and New Zealand context. In this article we outline recent progress intended to address this gap. A recent book chapter by Simpson [8] explains that core competencies in fetal surveillance could perhaps be assessed using a sample of 25 to 50 multiple-choice items. While this range of items might adequately sample core content in the domain, it is not guaranteed that acceptable (industrially defensible) psychometric quality will be attained. Simpson refers to an existing multiple-choice assessment in the United States context, developed and administered by the National Certification Corporation (NCC). The NCC examination consists of 100 scored items and has been evaluated psychometrically using an item response theory approach [9]. We are not aware of any other criterion-referenced and psychometrically validated assessments of knowledge underpinning core competencies in fetal surveillance. Owing to differences in EFM guidelines between certain countries and regions, it is appropriate to establish a separate but similarly validated assessment program in the Australia and New Zealand context.

### Psychometric validation

The validity of an assessment can be demonstrated by addressing three issues [10]:

...*there are certain critical abilities necessary for effective performance, and that individuals who lack these abilities will not be able to perform adequately in practice; individuals scoring low on the examination lack knowledge underlying these critical abilities and will not be able to practice in a safe and effective manner; and the examination can be designed to accurately identify the point at which the knowledge, skills, and abilities demonstrated on the examination are most indicative of the candidate's ability to practice in a safe and effective manner*.

The importance of fetal surveillance in society and the necessity for competent practitioners is highlighted in the preceding section. Within the domain of factual and conceptual knowledge that underpins fetal surveillance there exists a hierarchy of competencies that can be identified by subject-matter experts. This hierarchy is presented in Table 1. Herein the first assumption outlined in the previous paragraph is well-founded. The latter two assumptions require a methodology for linking performances on an assessment with an a priori specified description of the domain-specific knowledge and skills implied by those performances. One suitable approach is the implementation of a Rasch model analysis applied within the framework of criterion-referenced assessment design [11]. This approach has been applied in several contexts and is demonstrated in this article.

**Table 1:** Hypothesised competency levels for the FSEP assessment variable

**RANZCOG FSEP Expert a priori specified competency levels**	
**Level 1**	*CTG use under supervision*. • Understanding the basic physiology of fetal heart rate control • Being able to recognise what is normal and physiologically correct • Being able to recognise an abnormal CTG or heart rate pattern • Knowing who to call for assistance • Being able to set up and apply a CTG • Know the principles of conservative management and be able to apply these under supervision
**Level 2**	*Recognising trends in FHR Patterns and the implications of those trends*. • Being able to perform as an independent practitioner, with access to senior/supervisory staff • Being able to recognise and manage an abnormal CTG and appreciate the underpinning physiology • Having a detailed knowledge of indications for EFM • Appreciate the different modes of fetal monitoring and the indications and implications for each • Appreciate the implications of different management strategies • Being able to provide basic education for Level 1 practitioners

**Level 3**	*Recognising errors in the interpretation and management of CTG abnormalities, and the implications of those errors*. • Being able to instruct and teach others in EFM and fetal surveillance • Have a detailed knowledge of the normal and abnormal CTG, including the underpinning physiology • Being able to recognise and manage unusual abnormalities • Be able to manage multiple cases in a range of circumstances and understand the implications of those actions • Being able to nominate other investigations as required and understand their limitations and implications • Circumstances for referral and applied systems

### Reliability considerations

Reliability requirements for high-stakes assessments are often referenced to *The Standards for Educational and Psychological Testing*[12] published by the American Educational Research Association (AERA), the American Psychological Association (APA) and the National Council on Measurement in Education (NCME). These requirements are described in terms of indices of internal consistency such as the KR-20 and the Cronbach Alpha [13]. The standards and other authors [14,15] state that values of 0.9 are minimally acceptable for assessments used to make high-stakes decisions about individuals.

Other conceptions of reliability are also available to assessment designers. In the context of Item Response Theory (IRT), separation indices may take the place of internal consistency indices. Separation indices can also define the number of statistically different performance strata that the test can identify in the sample. For instance, a separation reliability of 0.8 is deemed sufficient for a test with three (equally distributed) levels and two corresponding cut-points [16]. This definition is only used as a guide in this context, since cut-points will not be constrained to cover equal intervals of the metric. Instead, cut-points will be assigned to locations on the metric where knowledge and skills, embedded in clusters of items, change substantively.

As a general rule, reliability indices are favourably impacted by larger numbers of assessment tasks or test items. Other considerations include the targeting of relative item difficulties to the abilities of the candidate population, and the statistical quality of the test items themselves.

The assessment instrument under analysis in this article is intended as a precursor to an extended test form that could be used to make high-stakes decisions about practitioners. This article is important for monitoring the development of the instrument by evaluating particular aspects of the 40 item trial test form. These aspects include item performance, item domain coverage, construct validity and reliability measures. By addressing each of these important test characteristics at the trial stage, development of a valid and legally defensible assessment instrument can be informed in a maximally efficient way. For instance, information regarding the nature of new items that are required, the anticipated increase in test length required, and the formalisation of an assessment blueprint are all attained as part of this analysis. Further, through the use of visually interpretable Rasch analysis output, expert panellists were able to make initial inferences about appropriate pass standards for the purposes of the assessment. Establishing a final pass standard will be undertaken using a documented pass standard methodology as a future exercise.

### Test blueprint

The fetal surveillance domain embeds particular knowledge and skills that are necessary for safe and effective practice at various levels of responsibility. These components of knowledge and skill can be separated into meaningful classifications in two ways: firstly, in terms of the type of content or context to which the knowledge or skills apply (e.g. definitions, applications of physiology definitions), and secondly in terms of the relative sophistication of the knowledge and skills with regard to their role in practice. A preliminary blueprint for the RANZCOG FSEP MCQ assessment is provided in Appendix A.

### Test calibration

IRT describes the relationship between a person's ability on some latent trait (measured by a test) and the person's observed responses to items on that test [17]. IRT enables construction of scales where both items and persons are assigned values representing their respective difficulty and ability. These values are mapped onto a common metric and are often presented visually on a variable map.

A commonly applied IRT model is the simple logistic Rasch model [18]. This model is suitable for scaling dichotomously scored data such as that for the RANZCOG FSEP assessment [19]. While other, more complicated variants of the Rasch model exist, they were not deemed necessary for the purposes of this study. For a more detailed account of IRT, the reader is encouraged to refer to Hambleton, Swaminathan and Rogers [20].

## Methods

### Sample

Data were collected from 877 practitioners undertaking education through the FSEP. The target population included all health professionals undertaking fetal surveillance. The sample comprised practitioners from a range of job classifications including trainee midwives, medical residents, midwives, obstetric registrars, general practitioners and specialist obstetricians, among others. Ethics approval for the analysis of item responses was granted through the Melbourne Graduate School of Education under application number 0608717.

### Item analysis

Both Classical Test Theory (CTT) and Rasch model statistics are reported in this study. The item analyses served primarily to identify any items or distractors that were performing poorly from a statistical standpoint. It was intended that these items might then be subjected to qualitative review and potentially amended (if not discarded) prior to redeployment in subsequent administrations. Further, the results from the item analyses were to be used to comment on the statistical performance of the FSEP assessment forms as a whole, particularly with regard to Rasch model fit and measures of test reliability.

### Establishing validity using Rasch modelling

Wright and Masters [16] showed that separating the items and identifying the skills underpinning each item could define the variable underpinning the test. They argued that the separation index provides evidence of construct validity. As such, the item separation index becomes one of the very few empirical indices of validity. Items that cluster together do not provide sufficient information about the variable to allow interpretation. However, if a sequence of clusters can be identified and each has a cohesive and interpretable meaning, the variable can be clearly identified. Once items have been calibrated along the variable, they can be interpreted in terms of the item writers' intentions. To achieve this, a skills audit is undertaken.

Wright and Stone [19] demonstrated how test items can be used to define a variable and how to operationally define that variable. Griffin [11] showed how the analysis can lead to a criterion referenced interpretation of an underlying construct. They each showed that a logistic calibration provides person-free estimates of item difficulty and that an examination of the spread of the difficulty values gives an indication of the direction of the examinee development defined by the latent variable. Hence, the test design task is to identify a set of items linked to a teaching curriculum that fit the Rasch model and demonstrate person-free difficulty values with consistent directional spread across sub-groups.

Consideration of other indices throughout Rasch model applications is also important. Examination of model fit can provide information about how justified it is to measure the underlying construct with the particular set of items chosen [19]. Good fit to the model suggests that the items are measuring the same one-dimensional construct, in other words, the assessment has construct validity. However, fit indices are not always sensitive to departures from unidimensionality. Instead, while working within the Rasch paradigm, it is more appropriate to evaluate dimensionality with principal components analysis (PCA) of the Rasch residuals [21]. The rationale for this is that after the "Rasch factor" has been extracted (in an attempt to account for all variation in the data) only standardised residuals equivalent to random, normally distributed noise should remain. PCA of these residuals should therefore not recover any meaningful patterns leading to identification of other factors unaccounted for by the extracted "Rasch factor". Small eigenvalues (less than 3) and low levels of explained variance (less than 5%) for the first residual component provide initial (but not equivocal) criteria in support of unidimensionality [21]. A more sensitive test recommended by Smith [22] and now commonly applied [21,23,24] separates items that load positively on the first residual factor from items that load negatively onto the first residual factor. Person scores (measured in logits) are then estimated using these item subsets, and after scale equating, scores from the two item subsets can be compared using paired t-tests with a consideration of the standard error of measurement for each score. The proportion of significantly different scores across item subsets is then calculated, with proportions of 5% or less being considered an indication of unidimensionality.

When the item pool meets the first mentioned criterion (fit to the model) and the scale meets the second mentioned criterion (unidimensionality), the test is considered capable of providing evidence that the target variable was being measured and that it is valid to examine differences in achievement (ability) across sub-groups. Analyses that seek to explain these differences in terms of professional practices, position, context or other variables of interest are then appropriate.

**Figure 1:**
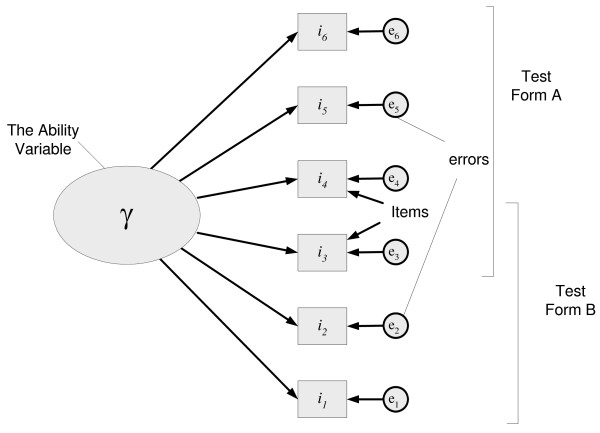
**Relationship between items, errors and the latent variable**.

The nature of the underlying variable and its use is important and preferred to a specific focus on each of the individual items. Embretson and Reise [25] showed that the overall measurement is made up of three parts. These were the underlying or 'latent' variable, the items or 'manifest' variables and the error associated with each of the items. Each individual item can be considered to measure a range of things, some of which are related to the latent construct, but they each measure other things as well. The extent to which these other things influence the measure is related to the reliability of the test. The extent to which the items or manifest variables relate as a set to the latent variable emphasises the validity of the test and the interpretability of the construct. The test instrument is always constructed to measure ability and difficulty on the latent construct, not on the individual items. In this sense, the nature and scope of item selection is unimportant provided that the item set is sampled to provide appropriate domain coverage. Figure 1 shows the relationship between the latent construct, the manifest variables and the error terms or noise in the measurement of the construct. It also shows that, when the items are sampled from a properly defined domain, sub-samples of items can equally be used as parallel test forms still representing the domain and still capable of mapping both item difficulty and person ability onto the latent construct. This enables an assessment program to maintain pass standards across administrations where test takers and items differ in relative ability and difficulty respectively. This is one of the key advantages of Rasch modelling over CTT [26].

Both the Quest [27] and ConQuest [28] software packages were used to obtain fit and separation indices and to produce visual mappings of the item and person locations.

**Figure 2:**
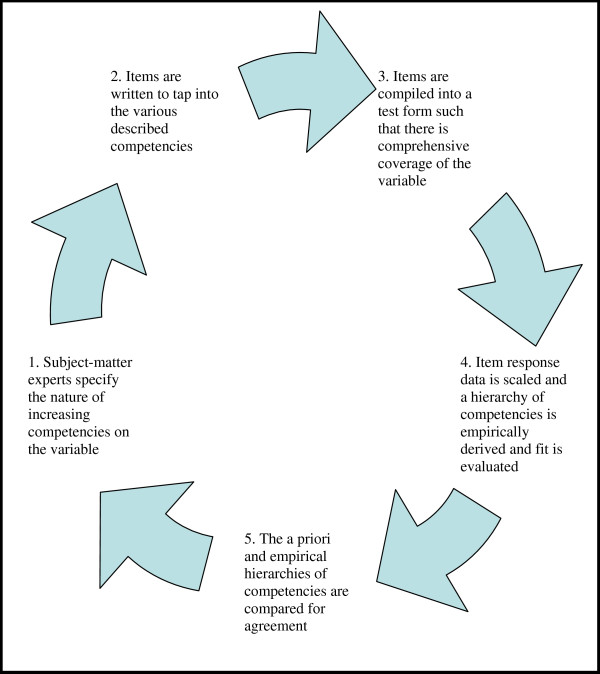
**Schema of the construct validation methodology**.

Once it has been established that the measured variable has direction and can define several statistically separate levels, the extent to which the variable meets the intentions of the assessment developer needs to be determined. If the items in the assessment adequately address the underlying variable intended by the assessment developer, then those items should have adequate fit to the model. The extent to which the set of items defines the variable, as described above, and fits the model is a measure of how well they provide construct validity, in other words, how well they measure a single, interpretable, underlying trait. The methodology for developing evidence of construct validity can be depicted as shown in Figure 2.

A second analogous set of questions relates to the extent to which the persons undertaking the assessment are distributed along the variable defined by the items. If the persons are clustered at extremes of the variable we have a "ceiling" or "floor" effect – the difficulty levels of the items that define the variable do not match the ability measure of the persons being assessed. Where persons are clustered too closely together, any inferences drawn from the assessment about practitioners' achievement may be compromised. Statistics are derived in the same way as the item statistics and include the person separation and potentially the person fit measures. These statistics can be produced using the Quest [27] software package.

The person measures provide additional information about the construct since they describe the extent to which the sample of test takers has responded in anticipated ways. If the responses of the practitioners do not provide good fit, or the separation of persons along the variable is poor, it suggests that the assessment does not adequately represent the ability and understanding of the sample. It thus provides one approach to concurrent validity [16]. Further, where separation is poor, additional items may be written to increase separation in subsequent administrations.

## Results

### Described competence levels

Prior to developing and administering the assessment described in this article, it was necessary to produce an initial assessment specification. A panel of eight subject-matter experts composed a hierarchy of three ordered competency levels (see Table 1). These were intended to capture the range of knowledge and skills required for conducting safe and effective practice across all levels of responsibility. They were also used to guide item construction when used in conjunction with a blueprint of content strands.

### Treating missing responses

Missing responses, of which there were relatively few (see column *missing *in Table 2), were treated as incorrect. This seemed reasonable given the proposed use of the FSEP instrument as a high-stakes regulatory assessment.

**Table 2:** FSEP assessment classical and Rasch item statistics (n = 877)

*Qn*	*ID*	*p*	*sd*	*d*	*r*	*a*	*A* *(%)*	*A* *(pt bis)*	*B* *(%)*	*B* *(pt bis)*	*C* *(%)*	*C* *(pt bis)*	*D* *(%)*	*D* *(pt bis)*	*missing (%)*	*Logit*	*Infit*
1	1002	0.88	0.33	0.25	0.30	0.79	5.59	-0.18	88.58	0.25	2.10	-0.15	3.26	-0.06	0.47	-1.22	1.00
2	1006	0.87	0.34	0.30	0.34	0.79	2.80	-0.18	3.26	-0.16	88.11	0.30	5.59	-0.16	0.23	-1.19	0.99
3	1007	0.57	0.50	0.36	0.29	0.79	19.11	-0.25	6.29	-0.11	58.04	0.36	16.55	-0.14	0.00	0.55	0.99
4	1008	0.89	0.31	0.10	0.19	0.80	1.40	-0.03	90.44	0.10	7.46	-0.08	0.47	-0.09	0.23	-1.46	1.06
5	1009	0.72	0.45	0.34	0.30	0.79	15.38	-0.31	1.63	-0.06	72.73	0.34	10.26	-0.10	0.00	-0.2	0.98
6	1003	0.85	0.35	0.37	0.39	0.79	86.48	0.37	12.12	-0.29	1.17	-0.23	0.23	-0.12	0.00	-1.1	0.95
7	1010	0.6	0.49	0.37	0.30	0.79	61.07	0.37	11.19	-0.28	25.17	-0.17	2.56	-0.10	0.00	0.44	0.98
8	1011	0.68	0.47	0.41	0.36	0.79	1.40	-0.09	68.76	0.41	5.83	-0.29	24.01	-0.26	0.00	0.29	0.98
9	1012	0.49	0.50	0.37	0.29	0.79	34.03	-0.21	10.96	-0.13	5.13	-0.20	49.88	0.37	0.00	1.06	1.01
10	1013	0.56	0.50	0.36	0.29	0.79	0.70	-0.04	39.39	-0.33	3.50	-0.06	56.18	0.36	0.23	0.56	0.98
11	1014	0.68	0.47	0.25	0.21	0.80	3.73	-0.05	68.30	0.25	1.63	-0.06	26.11	-0.21	0.23	0.31	1.06
12	1015	0.78	0.41	0.21	0.21	0.80	79.25	0.21	19.35	-0.20	0.93	-0.06	0.00	NA	0.47	-0.55	1.03
13	1016	0.71	0.45	0.39	0.35	0.79	5.59	-0.20	19.11	-0.24	71.79	0.39	3.26	-0.19	0.23	-0.16	0.94
14	1017	0.58	0.49	0.26	0.21	0.80	31.93	-0.11	5.83	-0.14	58.97	0.26	3.26	-0.25	0.00	0.55	1.02
15	1018	0.47	0.50	0.10	0.05	0.80	20.05	0.02	4.90	-0.17	27.27	-0.03	47.32	0.10	0.47	1.18	1.12
16	1019	0.87	0.34	0.33	0.36	0.79	6.99	-0.24	2.10	-0.10	1.63	-0.09	87.65	0.33	1.63	-1.16	0.98
17	1020	0.9	0.30	0.21	0.29	0.79	2.56	-0.14	91.38	0.21	2.80	-0.06	2.33	-0.12	0.93	-1.52	1.02
18	1021	0.7	0.46	0.43	0.39	0.79	10.72	-0.18	2.80	-0.28	15.62	-0.24	70.63	0.43	0.23	-0.05	0.95
19	1022	0.76	0.43	0.42	0.40	0.79	0.93	-0.10	77.39	0.42	19.35	-0.31	2.33	-0.29	0.00	-0.4	0.96
20	1023	0.91	0.28	0.26	0.35	0.79	1.86	-0.16	92.54	0.26	3.03	-0.14	2.56	-0.15	0.00	-1.91	0.98
21	1024	0.87	0.33	0.21	0.26	0.79	4.90	-0.06	88.34	0.21	3.73	-0.07	2.10	-0.16	0.93	-1.22	1.01
22	1025	0.75	0.43	0.32	0.30	0.79	75.99	0.32	5.13	-0.06	9.56	-0.25	8.16	-0.11	1.17	-0.22	0.99
23	1026	0.78	0.42	0.35	0.33	0.79	6.29	-0.28	78.55	0.35	4.20	-0.15	10.26	-0.11	0.70	-0.52	0.98
24	1027	0.54	0.50	0.03	0.00	0.80	54.55	0.03	37.76	0.02	3.26	0.00	3.26	0.00	1.17	0.87	1.15
25	1028	0.92	0.27	0.30	0.39	0.79	4.43	-0.21	93.47	0.30	0.70	-0.04	0.47	-0.08	0.93	-1.95	0.99
26	1029	0.86	0.35	0.32	0.35	0.79	87.18	0.32	6.53	-0.13	4.20	-0.27	1.63	-0.08	0.47	-1.11	0.98
27	1030	0.53	0.50	0.32	0.25	0.79	53.61	0.32	32.40	-0.11	6.06	-0.30	7.23	-0.09	0.70	0.89	0.99
28	1031	0.78	0.41	0.40	0.38	0.79	79.25	0.40	3.73	-0.03	2.10	-0.15	13.99	-0.33	0.93	-0.6	0.95
29	1055	0.29	0.46	0.37	0.28	0.79	29.84	0.37	1.17	-0.08	11.89	0.00	56.18	-0.28	0.93	1.78	0.98
30	1033	0.85	0.35	0.42	0.43	0.79	1.40	-0.12	7.46	-0.35	4.20	-0.13	86.48	0.42	0.47	-1.09	0.94
31	1062	0.89	0.31	0.17	0.25	0.79	1.63	-0.21	2.80	-0.02	4.43	-0.04	90.44	0.17	0.70	-1.39	1.02
32	1035	0.2	0.40	0.10	0.05	0.80	8.16	0.04	20.05	0.10	41.26	0.04	29.60	-0.13	0.93	2.44	1.08
33	1036	0.44	0.50	0.27	0.20	0.80	32.87	0.01	13.75	-0.23	44.99	0.27	6.76	-0.23	1.63	1.18	1.02
34	1037	0.76	0.43	0.33	0.31	0.79	3.73	-0.17	8.62	-0.12	76.46	0.33	10.26	-0.21	0.93	-0.35	1.00
35	1001	0.35	0.48	0.27	0.19	0.80	46.15	-0.15	35.43	0.27	4.43	-0.04	12.82	-0.12	1.17	1.78	1.00
36	1038	0.69	0.46	0.45	0.40	0.79	20.28	-0.36	5.59	-0.06	69.93	0.45	3.73	-0.19	0.47	-0.04	0.92
37	1039	0.36	0.48	0.18	0.11	0.80	51.98	0.02	36.36	0.18	2.80	-0.19	8.16	-0.18	0.70	1.57	1.08
38	1040	0.39	0.49	0.41	0.32	0.79	39.39	-0.13	39.86	0.41	15.15	-0.25	4.90	-0.17	0.70	1.4	0.95
39	1041	0.26	0.44	0.15	0.09	0.80	56.18	0.00	13.29	-0.10	3.73	-0.11	26.11	0.15	0.70	2.23	1.08
40	1042	0.63	0.48	0.39	0.33	0.79	7.23	-0.14	5.13	-0.24	64.10	0.39	23.08	-0.20	0.47	0.33	0.96

### Item Analyses

Classical statistics in addition to Rasch calibration estimates are presented for all items in Table 2. For each item, the summary statistics were as follows:

1. Item number in test form (Qn)

2. Item bank identification tag (ID)

3. The p-value represents the proportion of the sample with the correct answer. This is the mean item score (p)

4. The item standard deviation (sd)

5. The classical item discrimination (d) which should be at least 0.2 but preferably closer to or in excess of 0.4 [29]

6. The correlation between the item score and the test total score (r) which should be at least 0.3 [30]

7. The alpha reliability estimate of the test has then been presented if the item were to have been omitted from the test (a)

8. For each item distractor follows a sequence of percent-correct scores and point biserial correlation values, the latter of which should be positive corresponding to correct responses and negative corresponding to incorrect responses

9. The percentage of missing responses is then presented

10. The item difficulty (Logit)

11. Infit mean square estimate (Infit) which should be within the range 0.8 to 1.2 [31].

### Item performance

The median item discrimination value was 0.32. The lower and upper bounds of the corresponding interquartile range were 0.24 and 0.37. This indicates that most items discriminated positively and effectively. Modest values were anticipated for the items which were found to be relatively easy. However, seven items had classical item discrimination values between 0.03 and 0.2. It was determined that these items warranted a qualitative review. In particular, items 4, 15, 24 and 32 (with discrimination values of 0.1 or less) were to be omitted from the empirical derivation of described competency levels and the residual-based evaluation of scale dimensionality. The standard error of measurement associated with each logit value was approximately 0.07 logits. This was deemed sufficiently small for the purposes of this study.

### Test performance

Several key test parameters were also tabulated from the CTT and Rasch analyses so that any weaknesses concerning the assumptions and requirements of the assessment could be identified. These are summarised in Table 3.

**Table 3:** Test statistics from CTT and IRT analyses of the 40 item RANZCOG FSEP trial instrument

**Parameter**	**Value/s**	**Comments**
Number of candidates, N	877	This was considered adequate for the purposes of a trial analysis for informing refinements to the extended test instrument. The resultant standard error of measurement for item difficulty parameter estimates was small at 0.07 logits.
Cronbach's Alpha	0.80	This was promising given the use of some underperforming items and a shortened test form. However, the intention will be to increase test length, item quality and test targeting to achieve a value in excess of 0.9.
Item Separation Reliability	1.00	This provides evidence that item parameter estimates are adequately separable and varied.
WLE Person Separation Reliability	0.71	Much as for Cronbach's Alpha, this value will need to be increased with the introduction of additional quality, targeted items. High Person Separation values are of particular importance when determining the number of performance levels and corresponding cut-scores that can be specified for a single assessment.
Mean Test Score (and Standard Deviation)	26.7 (5.7)	These values (mean and standard deviation) suggest that the test is not too easy or too difficult for the practitioner population and is not subject to "floor" or "ceiling" effects.
Mean Item Infit (and Standard Deviation)	1.00 (0.05)	These values (mean, and in particular, standard deviation) support the assumption that the FSEP instrument measures a single, unidimensional construct. This was important for justifying continued use of the Rasch-based methodology.
Proportion of t-tests outside 95% confidence interval	7.75 (5.98 – 9.52)	This proportion indicates a slight departure from unidimensionality. Other item and test statistics did not detect this departure. This technique will be repeated once further item revisions have been carried out. Items with strong opposite loadings will be compared through qualitative review and evaluated against the intended construct.

The reliability and fit values in Table 3 provide initial evidence that the test was measuring a single dominant variable and that a single dominant latent variable underpinned the set of items. The PCA of Rasch residuals produced a first residual component with an eigenvalue of 1.65 explaining 4.65% percent of variance. These figures suggest that no additional structures were obviously apparent in the FSEP scale. The analysis of the proportion of non-invariant person scores revealed a slight departure from unidimensionality. The proportion of differences was calculated as 7.75% (68/877) with a 95% confidence interval slightly in excess of the 5% critical value (see Table 3). A significant change in fewer than 59 individual scores was required to accept the hypothesis of unidimensionality (and the local independence of items). This result may improve following a review of the remaining underperforming items. This result will also prompt a qualitative review of differences between the cognitive demands of the positive and negative loading items in light of the intended construct. Overall the test statistics indicated that the test could reasonably separate candidates on the basis of ability (i.e. that it possessed acceptable criterion validity) as well as demonstrating construct validity. The priority emerging from these statistics was that the number of items would still need to be increased in the move to high-stakes applications of the assessment.

### Variable Maps

**Figure 3:**
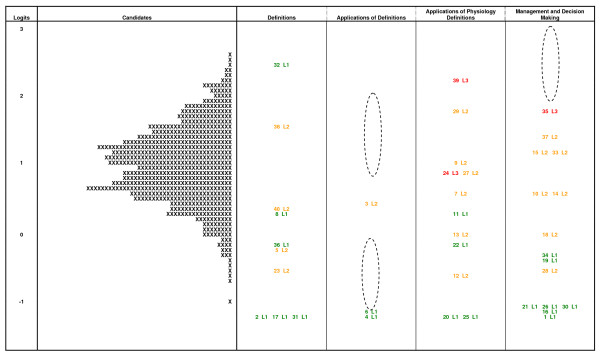
**Variable map from the initial assessment data**.

Many of the characteristics of the test can be identified from what is referred to as a *Variable Map*. This has been presented for the RANZCOG FSEP test in Figure 3. The chart has several sections to it. Working from the left of the figure the first characteristic of the chart is a scale that ranges from approximately -2.0 to +3.0. This is the logit scale and is the metric of the Rasch model analysis that enables person ability and item difficulty to be mapped conjointly. The distribution of pupil ability is presented next and each 'X' represents 1.3 candidates. It clearly shows that the range of ability is quite broad.

The next component of the chart is the distribution of items illustrating their relative difficulty. It can be seen that these ranged from about -1.5 to 2.5 logits. Codes are used to describe and identify each item. In this program, colour coding and level labels (i.e. L1, L2 and L3) were also incorporated to differentiate between items that were intended to tap into the different hypothesised levels. This highlighted items that were perceived by the practitioner population as being more difficult or easier than the item writing panel had envisaged. For example, item 32 (labelled *32 L1*) was found to be more difficult than the expert committee had expected. It was therefore suggested that the item be qualitatively reviewed so that an explanation for this discrepancy might be established.

In Figure 3 the chart illustrates how the items on the test were divided into blueprinted content domains. It can be seen that the distribution of item difficulties in each of the domains varies and does not necessarily cover the range of candidate abilities across all blueprint columns. For some content domains (such as *Definitions*) this might be appropriate but for others it may be necessary to introduce new items.

It can also be seen that there are gaps, as highlighted by the ellipses. This is important information for two reasons: firstly, subsequent item and test development can be informed by targeting new items at the location of the gaps, and secondly, these gaps indicate regions in which there is minimal information about candidate skills. Appropriately writing and seeding new items to fill these gaps increases the capacity of the test to discriminate between candidates of similar abilities on the basis of knowledge and skills matched to the regions where such gaps emerge [19].

### Interpreting the test: Competency levels

In addition to ability measures, other measures related to educational outcomes were derived from the data. One of these has been referred to as the competency levels and relates directly to the definition of criterion-referenced interpretation of tests. Combining the ideas of criterion-referenced interpretation with item response modelling directly links the position of a person or an item on a variable (as shown in the variable map) to an interpretation of what a practitioner, or groups of practitioners, can do, rather than focussing on a score or the performance relative to a percentage or a group. It also orients the use of the test data towards substantive interpretation of the measurement rather than reporting a score or grade. The procedure gives meaning to test scores and is used here to derive the substantive interpretation of the levels of increasing competence.

It can be seen from the variable map in Figure 3 that several items grouped together at different points along the uni-dimensional scale. The major question was whether these clusters could be interpreted as having something in common. Each item was reviewed for the skills involved in responding to the item and it was a matter of substantive interpretation. The process required an understanding or empathy with 'how the practitioners think' when they are responding to the items.

**Figure 4:**
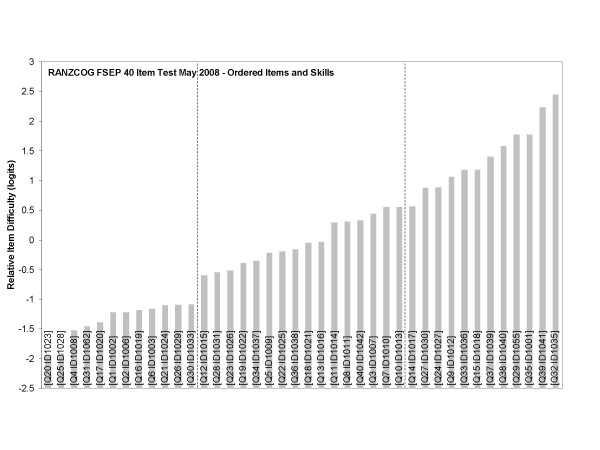
**Items ranked by relative difficulty with cognitive skill descriptions (preliminary item cluster cut-points shown as vertical dashed lines)**.

To assist in this procedure the logit values of the item difficulties were ordered according to increasing item difficulty. Each item was also analysed for the underpinning cognitive skill involved in obtaining the correct answer. The results of these analyses have been presented in Table 4 and Figure 4.

**Table 4:** Cognitive skills audit as carried out by the specialist panel

**Item**	**Logit**	**Cognitive skill and knowledge**
Q20 ID1023	-1.95	Demonstrating a knowledge of fetal heart rate control
Q25 ID1028	-1.91	Demonstrating an understanding of the physiology of fetal heart rate control
Q4 ID1008	-1.52	Application of definition to interpret trace characteristic (baseline fetal heart rate)
Q31 ID1062	-1.46	Knowledge of definition of hyperstimulation (physiology of) as per local guidelines
Q17 ID1020	-1.39	Knowledge of the causes of reduced baseline variability
Q1 ID1002	-1.22	Combine basic trace and physiology information to determine appropriate management decision within clinical context
Q2 ID1006	-1.22	Knowledge of definition of the baseline range as per local guidelines
Q16 ID1019	-1.19	Knowledge of indications for EFM as per local guidelines
Q6 ID1003	-1.16	Knowledge and application of a definition (baseline variability)
Q21 ID1024	-1.11	Recognition of common abnormal trace pattern
Q26 ID1029	-1.10	Recognition of appropriate/inappropriate indications for EFM as per local guidelines
Q30 ID1033	-1.09	Combine basic trace and physiology information to determine appropriate management decision within clinical context
Q12 ID1015	-0.60	Interpretation of CTG trace abnormaility (receptors)
Q28 ID1031	-0.55	Synthesising trace and physiology information to determine appropriate management decision within clinical context
Q23 ID1026	-0.52	Knowledge of CTG trace limitations (sensitivity/specificity)
Q19 ID1022	-0.40	Knowledge of indications (or lack of indications) for EFM as per guidelines
Q34 ID1037	-0.35	Recognising appropriate management for the given circumstance
Q5 ID1009	-0.22	Demonstrating an understanding of the definition of baseline variability
Q22 ID1025	-0.20	Understanding the relative importance of CTG trace characteristics (baseline variability)
Q36 ID1038	-0.16	Identification of correctly paraphrased physiology definition (acceleration)
Q18 ID1021	-0.05	Synthesising trace and physiology information to determine appropriate management decision within clinical context
Q13 ID1016	-0.04	Understanding the relative importance of CTG trace characteristics (baseline variability)
Q11 ID1014	0.29	Application of definition to interpret trace characteristic (baseline fetal heart rate)
Q8 ID1011	0.31	Identification of correctly paraphrased physiology definition (CTG baseline fetal heart rate)
Q40 ID1042	0.33	Recall of definitions of early decelerations
Q3 ID1007	0.44	Application of definitions to trace characteristics (baseline fetal heart rate and baseline variability)
Q7 ID1010	0.55	Synthesising trace and physiology information in relation to the fetal condition
Q10 ID1013	0.55	Synthesising trace and physiology information to determine appropriate management decision within clinical context
Q14 ID1017	0.56	Synthesising trace and physiology information to determine appropriate management decision within clinical context
Q27 ID1030	0.87	Synthesising trace and physiology information in relation to the fetal condition
Q24 ID1027	0.89	Synthesising trace and physiology information to determine fetal wellbeing within the clinical context
Q9 ID1012	1.06	Synthesising trace and physiology information in relation to the fetal condition
Q33 ID1036	1.18	Synthesising trace and physiology information to determine appropriate management decision within clinical context
Q15 ID1018	1.18	Synthesising trace and physiology information to determine appropriate management decision within clinical context
Q37 ID1039	1.40	Synthesising trace and physiology information to determine appropriate management decision within clinical context
Q38 ID1040	1.57	Application of definition to interpret trace characteristic
Q29 ID1055	1.78	Demonstrating a knowledge of the physiology of fetal heart rate control
Q35 ID1001	1.78	Synthesising trace and physiology information to determine appropriate management decision within clinical context
Q39 ID1041	2.23	Synthesising trace and physiology information to determine fetal wellbeing within the clinical context
Q32 ID1035	2.44	Understanding the definition of the baseline fetal heart rate

The question then arose that if the difficulty increased for sets of items, did the nature of the underpinning skill also alter? The two sets of information were then explored in unison. Natural breaks in difficulty were identified and then the items and the cognitive descriptions were examined to determine if a set with a common substantive interpretation could be found. Measurement error was also taken into consideration to determine whether breaks were statistically significant (see the PISA 2003 Technical Report published by the OECD for a description of this issue). A panel of eight subject-matter experts undertook this exercise. Together they identified the breaks in the variable and then offered the substantive interpretation of the levels of proficiency. These have been presented in Table 5.

**Table 5:** Empirically derived competency levels for the FSEP assessment variable

	**RANZCOG FSEP Empirically derived competency levels**
**Level 1**	To achieve correct responses at this level, participants need to be able to recognise what is normal in terms of the CTG. They also need to appreciate the physiology implicit in the normal FHR control. They need to know the definitions of the individual characteristics of a normal CTG. They need to appreciate the indications and contraindications for continuous electronic fetal monitoring (EFM). Participants need to be able to recognise low level abnormalities and the principals of conservative management.

**Level 2**	To achieve correct responses at level 2, in addition to the previous requirements, participants need to be able to recognise the uteroplacental and fetal physiology implicit in the more common CTG abnormalities. They need to be able to manage these abnormalities within a given context. They need to appreciate the limitations as well as the different modalities of EFM. They also need to recognise trends in CTGs and the implications of those trends. Some of the more basic skills from level 1 are linked to harder CTGs in these level 2 items.

**Level 3**	To achieve correct responses at level 3, participants need, in addition to the previous requirements, a detailed understanding and appreciation of the physiology of high risk as well as less common FHR abnormalities. Participants need to appreciate additional forms of fetal assessment and their applicability. At this level, practitioners also need to be able to recognise errors in information and interpretation. They need to be able to make high level management decisions based on accurate synthesis of complex information from a wide range of information sources.

### Establishing competency levels empirically

With a view to deriving described competency levels, initial analysis focused on the ordered item bar chart (Figure 4). Four or even five potential cut points or levels appear apparent where there is an abrupt change in relative item difficulty over groups of items. Closer analysis of the chart and of the discriminatory capacity of the individual items reveals just three distinct cut points. Equally, around each of these points there is an identifiable change in the relative cognitive skills required by participants.

In order to identify the cut points, analysis of the individual items was undertaken. As previously described, item performance (Table 2) revealed four questions with low discrimination values, requiring their removal from the item bank and exclusion from consideration in this exercise. Also, as previously discussed, the variable map (Figure 3) highlights questions which appeared more or less difficult than the subject matter experts had supposed. Closer inspection of these items typically revealed simple but fundamentally important concepts ensconced in inappropriately complex questions. Bearing the limitations of these individual items in mind and their relative positioning on the ordered item bar chart, a direct comparison with the skills audit demonstrates the three cut points previously alluded to.

Up to cut point 1 (ID1033) [Level 1], the bulk of the items are simple questions based around simple concepts; such as recall of definitions and application of those definitions. There is some basic physiology of fetal heart rate (FHR) control, generally as applicable to the normal CTG. Most of these early items do not have a CTG example attached or clinical scenario and therefore little synthesis of information is required. Of the two questions which do have a CTG attached, both require low level synthesis of "normal" CTGs to achieve the correct response.

From cut point 1, (ID1015) [Level 2], the items require a slightly higher understanding of the physiology of FHR control and application of that physiology to a given circumstance. Increasingly, these questions involve CTG/FHR abnormalities. There are some recall of definition items, with a few having an associated CTG and or a clinical scenario. Around half of these Level 2 items have a CTG and/or clinical scenario and increasingly these items require moderate level synthesis.

From the second cut point (ID1017) [Level 3], almost all the questions have an associated CTG and clinical scenario. Of the two which do not, one is for removal and the other requires rewriting to improve its discriminatory capacity. Increasingly, these items require high level synthesis of clinical scenarios, with an abnormal CTG and its implicit physiology to determine appropriate management. Increasingly the CTGs associated with these level 3 items involve less common but high risk examples of CTG abnormalities.

Herein the skills within each cluster were paraphrased to produce global proficiency level descriptors. This process utilised the range of sources of evidence described previously: the Rasch variable map (Figure 3), the bar graph of items ranked by relative difficulty (Figure 4), and the descriptions of cognitive skills embedded within the items (Table 4). The relative merit of each item's statistical performance was also noted by making use of Table 2. These pieces of evidence, when analysed in unison, enabled a methodology for locating and defining the substantive skills within different bands of the measurement

### Evaluating competency level agreement

Qualitatively evaluating the agreement between the contents of Table 1 and Table 5 constituted a key step in justifying the extent to which construct validity had been achieved (beyond the indications of the reliability and fit statistics).

The number of levels identified empirically for this test was the same as the number of levels hypothesised by the panel of experts. While this condition is not necessary to uphold consistency between hypothesised and derived levels, it does simplify evaluation of their correspondence. The content and direction of the empirically derived levels from the skills audit follows the same direction, structure and content as the hypothesised directions mooted by the expert panel in the initial development of the test. In fact, the link between the hypothesised and derived constructs is clear. The empirical data strongly supports the planned hypothesised direction of the test. Given a set of circumstances, the test can be used, with confidence in line with measurement error considerations, to identify the level of competence of the practitioners who have been assessed using the instrument.

The order and content of the knowledge involved in the levels identifies the typical level of competence of the practitioners. It is also evident that the distribution of practitioners over the variable covers the full range. The expert panel will use these results to make decisions about how to treat this data and what to do regarding the use of the performance levels. More importantly, those involved in providing the education program can use the distribution of practitioners over levels of competence to identify points of intervention for supplementary training and advice to practitioners regarding their likelihood of being successful in a retest situation after additional training.

## Discussion

We have reported here the steps undertaken to develop a valid and reliable assessment tool for application in a high stakes clinical environment. In doing so, we present only the second such assessment in fetal surveillance education, highlighting the challenges in its development, the limitations of the developed assessment and directions for future development. The Rasch-based validation methodology demonstrated here provides support that the initial test form measures the intended construct. There are however a number of quantitative psychometric targets that the instrument does not meet in its current form. Seven items were found to discriminate poorly, the test had moderate reliability, and a small deviation from unidimensionality, a condition for interval measurement, was detected. These issues will each require rectification in moving to an operational assessment suitable for making high-stakes decisions about individual practitioners.

**Figure 5:**
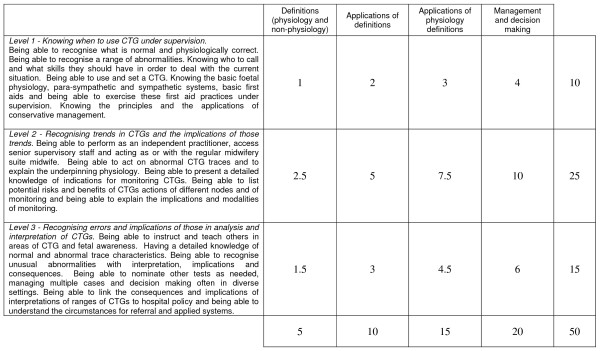
**Draft blueprint for proposed 50 item test (additional content lists are available to item writers to elucidate each of the four column categories)**.

In revisions of the test it will be necessary to do several things. First, additional items are needed to address gaps in the variable and to increase test reliability. However there is a trade-off between test length and the time required to administer and score the test. Resolving this issue is both a matter of test design and pragmatics of test administration. The important point is to achieve the greatest accuracy with the most efficiency. In order to maintain a relatively short administration time in this context, one or more 50 item test forms will be compiled and administered in place of the current 40 item test form as a next step. Item writing will be undertaken to address gaps in the variable and the blueprint. The location on the variable and the relative difficulty of these items is indicated in the variable map depicted in Figure 2 and the test blueprint in Figure 5.

Given information about item performance, identifying those items where distractors are not performing as well as expected and those items where discrimination is less than satisfactory would provide information that would improve the quality of the test. In reviewing each of the item distractors there is the option to either improve or remove faulty distractors from the items. The tendency to write four alternatives for every item at times reduces reliability. Maximum reliability in relation to the number of distractors has been achieved using three alternatives including the correct answer [32]. It may be that many of the items can be improved by removing the weaker alternatives.

In some cases item discrimination may be low because of the high overall ease or difficulty of the test item. Using item discrimination as an indicator of an acceptable item is not always successful when using item response modelling or latent trait applications of test theory. It is important that the items are distributed by difficulty over the range of ability of the target group of candidates. This means that there must be some very difficult items and some very easy items such that all candidates are matched to items regardless of the candidate ability. The consequence of this is that there will be items at the extremes of difficulty and facility and these items will have low correlations with total score and hence low discrimination. But they provide important information in targeting the ability level of candidates at those points on the latent variable.

The targeting of candidate ability using items of variable difficulty is a benefit of the latent trait approach to test development and interpretation. In classical test theory instructions to item writers usually indicate that all items should have a difficulty level of approximately 0.5. This meant that the items tended to cluster around the mean of the latent variable and the candidates at the extreme upper and lower levels of the variable were not matched by any items. This in turn meant that the test user could not identify points of intervention for all possible retraining programmes based on candidate's location on the underlying latent trait. It usually led to an item by item analysis of the candidate's performance and explicit teaching of item content. This is not a satisfactory approach to the use of assessment data for decision-making or for identifying training needs.

It is important to note that this article describes an evaluation of two components of the whole validity argument – content validity and construct validity. Content validity is addressed by the subject-matter expert group who conducted what is termed a Job Task Analysis whereby all relevant skills and competencies for the domain are specified. Considering the relative complexity of the skills and competencies culminates in an assessment blueprint to guide item and test construction. Construct validity is in this case addressed by comparing the hypothesised ordering of skills and knowledge with the empirically derived ordering of skills and knowledge arising from the Rasch scaling of item responses to a preliminary test form. The notion of predictive reliability, where performance on the assessment is compared with future performance in practise, is not within the scope of this paper. Nor is an evaluation of concurrent validity, where practitioner groups would be expected to be separated along the scale in accordance with other indicators of competence such as qualifications, relevant experience or performance on auxiliary but related assessment tasks. These evaluations are earmarked as future validation studies once subsequent test forms begin to meet the psychometric targets outlined in this article.

One additional validity consideration relates to the presence of bias in the items across distinguishable subgroups within the target population. In the context of the RANZCOG FSEP target practitioner population, several population subgroup classifications can be made based on qualification and or professional experience (for example, trainee midwife, midwife, registrar, general practitioner, obstetrician). IRT can be used to investigate differential performance on items between practitioners of equal ability but from different population subgroups. Specifically, the technique of Differential Item Function (DIF) analysis can be applied to detect such bias at the item level [33]. DIF analysis is not reported in this paper, but it is a quality control component of the FSEP assessment program.

For the present, the FSEP test will remain as a supervised, paper-based assessment, rather than an online assessment. This is despite the potential of online forms to exploit more authentic item types, and also to facilitate remote access. Currently FSEP does not have the infrastructure in place to administer the tests online on a large scale in a secure manner that would safe-guard the question bank. It has always been intended that the education and testing provided by the FSEP will form the basis of a regular competency assessment. In this way the assessment is functioning as a risk management strategy for the institutions whose staff attend. The validity of the assessment in this high stakes format is therefore critical. An online format was thus deemed inappropriate at this time due to the threat of item exposure and the resources required for conducting online testing under supervision.

## Conclusion

This article demonstrates the application of the Rasch model to a multiple choice assessment of intrapartum fetal surveillance content knowledge. Early evidence of construct validity was demonstrated through the correspondence between the empirically derived interpretation of the variable with that posited a priori by subject matter experts. Evaluation of a number of quantitative indices of item and test quality revealed certain threats to validity such as weak discriminating items, moderate reliability and a slight detectable departure from scale unidimensionality. However, several potential methods are documented in this article as a basis for efficiently improving the quality of subsequent test forms. Further validation studies are also described for when the key targets of psychometric quality outlined in this article have been attained.

## Competing interests

The authors declare that they have no competing interests.

## Authors' contributions

NZ and PG conducted and interpreted the statistical analyses of items and test forms. MB coordinated the associated test development and validation program. This included assembling expert practitioner groups, and compiling and administering test forms. Both MB and EW led the item writing workshops and led discussions about critical practitioner competencies and educational outcomes in light of the relevant guidelines.

## Pre-publication history

The pre-publication history for this paper can be accessed here:


